# First attempt success of video versus direct laryngoscopy for endotracheal intubation by ambulance nurses: a prospective observational study

**DOI:** 10.1007/s00068-020-01326-z

**Published:** 2020-02-19

**Authors:** Wim Breeman, Mark G. Van Vledder, Michael H. J. Verhofstad, Albert Visser, Esther M. M. Van Lieshout

**Affiliations:** 1 AmbulanceZorg Rotterdam-Rijnmond, P.O. Box 4, 2990 AA Barendrecht, The Netherlands; 2 Trauma Research Unit Department of Surgery, Erasmus MC, University Medical Center Rotterdam, P.O. Box 2040, 3000 CA Rotterdam, The Netherlands

**Keywords:** Ambulance, Endotracheal intubation, Laryngoscopy, Prehospital, Video laryngoscopy

## Abstract

**Purpose:**

The aim of this study was to compare the rate of first attempt success of endotracheal intubation performed by ambulance nurses in patients with a Glasgow Coma Scale (GCS)  of 3 using video laryngoscopy versus direct laryngoscopy.

**Methods:**

A prospective cohort study was conducted in a single, independent ambulance service. Twenty of a total of 65 nurse-staffed ambulances were equipped with a video laryngoscope; a classic direct laryngoscope (Macintosh) was available on all 65 ambulances. The primary outcome was first attempt success of the intubation. Secondary outcomes were overall success, time needed for intubation, adverse events, technical or environmental issues encountered, and return of spontaneous circulation (ROSC). Ambulance nurses were asked if the intubation device had affected the outcome of the intubation.

**Results:**

The first attempt success rate in the video laryngoscopy group [53 of 93 attempts (57%)] did not differ from that in the direct laryngoscopy group [61 of 126 (48%); *p* = 0.221]. However, the second attempt success rate was higher in the video laryngoscopy group [77/93 (83%) versus 80/126 (63%), *p* = 0.002]. The median time needed for the intubation (53 versus 56 s) was similar in both groups. Ambulance nurses more often expected a positive effect when performing endotracheal intubation with a video laryngoscope (*n* = 72, 81%) compared with a direct laryngoscope (*n* = 49, 52%; *p* < 0.001).

**Conclusion:**

Although no significant effect on the first attempt success was found, video laryngoscopy did increase the overall success rate. Ambulance nurses had a more positive valuation of the video laryngoscope with respect to success chances.

## Background

Prehospital endotracheal intubation can be challenging. Obtaining sufficient training and exposure to safely perform endotracheal intubation in the field is one of the major challenges for paramedics or ambulance nurses, but also prehospital doctors who are not anesthesiologists [1].

Reported first attempt success rates of prehospital endotracheal intubation vary substantially between different Emergency Medical Services (EMS) systems. A meta-analysis by Crewdson et al. reported an overall success rate for intubation ranging from 62 to 100% for non-physicians [2]. Differences in training, experience and exposure with regard to advanced airway management are most likely the major contributing factors to the discrepancy between studies.

As multiple attempts for endotracheal intubation are associated with poor outcome for several patient categories, some have advocated the use of supraglottic airway devices instead of endotracheal intubation for non-physician prehospital airway management. It is hypothesized that these are easier to insert and their successful placement is less operator dependent when compared to endotracheal intubation [3, 4]. However, a major disadvantage of this approach would be a further loss of exposure to laryngoscopy and subsequent tube placement for prehospital healthcare workers.

It would, therefore, be useful if the impact of training and exposure on the success rate of endotracheal intubation could be lowered. One way to achieve this would be to simplify the procedure by enhancing visibility of the vocal cords by video laryngoscopy. Not surprisingly, several EMS organizations have implemented video laryngoscopy as the primary device for prehospital endotracheal intubation, aiming to improve the first attempt and/or overall success rate. Unfortunately, the current literature does not unanimously show that video laryngoscopy indeed increases the intubation success rate. A Cochrane review including 64 studies with 7044 participants did not show a difference in the first attempt and overall success rate between conventional and video laryngoscopy [5]. However, the majority of studies in this review included experienced anesthesiologists in a hospital setting. As ambulance nurses have much less experience with endotracheal intubation than anesthesiologists, it is questionable if these results can be translated to intubation by ambulance nurses.

Therefore, the aim of this study was to compare the rate of first attempt success of endotracheal intubation performed by ambulance nurses in patients with a Glasgow Coma Scale (GCS)  of  3 using video laryngoscopy versus direct laryngoscopy. Secondary aims were to compare the overall success rates and the time needed for the procedure, and to determine complications and technical issues of both devices.

## Methods

### Study design

A prospective, observational cohort study was conducted from November 6, 2015 to July 29, 2017. The study was exempted by the local Medical Research Ethics Committee (reference number MEC-2015-467) with waiver of the requirement for informed consent. The study is registered at the Netherlands Trial Registry (NTR6174; date 10-Oct-2016).

### Setting

This study was conducted by a single Emergency Medical Service (EMS) in Rotterdam (The Netherlands). A total of 150 ambulance nurses rotate over 65 advanced life support (ALS) vehicles. The EMS serves an area with 1.3 million inhabitants and responds to approximately 126,000 emergency calls annually. Each ALS unit is staffed by an ambulance nurse and an ambulance driver. The ambulance nurses are fully registered nurses; they must have obtained additional certification in intensive care, emergency care, or anesthesia nursing before they are allowed to apply for the ambulance nurse educational program. In addition to ‘on-the-job-training’, they also completed a 9-month ambulance educational program during which they are supervised by an experienced EMS teaching nurse. After successful completion of the training, ambulance nurses are legally authorized to carry out medical procedures according to the nationwide ambulance protocols based on provisional diagnoses from clinical signs, symptoms, and mobile point-of-care diagnostic tools.

For airway management, the ambulances are equipped with a direct endotracheal intubation device, bag-valve-mask ventilation, and supraglottic airway. Endotracheal intubation is only allowed in patients with a severe loss of consciousness (GCS = 3) due to a non-neurological etiology and in whom a non-drug-assisted endotracheal intubation can be safely performed. In all other cases, alternative airway devices or Helicopter EMS assistance for drug-assisted intubation is advised by the national ambulance protocol.

### Patient selection

Patients with a Glasgow Coma Scale of 3 who had no suspicion of a primary neurological etiology or traumatic brain injury resulting in a loss of consciousness and who required prehospital endotracheal tube intubation by an ambulance nurse were eligible. In case the exact age was not known on the scene, patients had to have an estimated age of 18 years or older. Patients who afterwards turned out to be below 18 years of age were removed from the analysis in retrospect. Patients for whom the ambulance nurse forgot to use capnography prior to the first intubation attempt or in whom capnography did not show carbon dioxide production on the capnogram due to technical issues were excluded.

### Treatment

A random sample of 20 ambulances was equipped with a video laryngoscope (McGrath MAC Laryngoscope). The devices were kindly made available during the study period by Aircraft Medical Ltd (Edinburgh, United Kingdom). These 20 ambulances also had the normal direct laryngoscope (Macintosh) on board. Prior to the study, all ambulance nurses attended a didactic and hands-on training session defining the study purpose, study protocol, and practical training in using the video laryngoscope. A short refresher instruction was given on the days the ambulance nurse was allocated to an ambulance equipped with a video laryngoscope. This way, they had experience with both the direct and video laryngoscope.

Blade size and endotracheal tube size were left to the judgement of the ambulance nurse. Both devices were used according to the supplier’s protocol. According to the national ambulance protocol, only two intubation attempts were permitted. In case the second attempt failed, the nurses could use another device or technique such as a supraglottic airway device or bag-valve-mask ventilation.

### Outcome measures and data collection

The primary outcome measure was first attempt success, which was defined as successful placement of the endotracheal tube in the trachea on the first intubation attempt. A normal-appearing wave form on the capnogram and a value on the digital numeric display confirmed the position of the endotracheal tube in the trachea. The secondary outcomes were overall success, time to successful endotracheal intubation, adverse events, technical problems, and return of spontaneous circulation. Overall success was defined as successful placement of the endotracheal tube within two attempts as allowed by the national ambulance protocol.

Ambulance nurses were asked to complete a case report form after each intubation attempt. The form ensured standardized data collection of intubation characteristics and also recorded patient and environmental factors that may have had an effect on the intubation.

### Sample size

The participating EMS performs approximately 500 endotracheal intubations annually. With 60 ambulances, each team performs 8.3 intubations annually. Equipping 20 ambulances with a video laryngoscope will, on average, result in 167 endotracheal intubations with a video laryngoscope and 333 with a direct laryngoscope. Preliminary screening suggested a 60% success rate using a direct laryngoscope. A 15% improvement was considered clinically relevant and would justify the financial commitment needed to equip all ambulances with a video laryngoscope. A two-sided test with an *α* level of 0.05 and a *β* level of 0.1 requires 125 and 200 intubations with a video and direct laryngoscope, respectively.

### Statistical analysis

Analyses were performed using the Statistical Package for the Social Sciences (SPSS, version 21, SPSS Inc., Chicago, IL, USA). Normality of continuous data was checked using the Shapiro–Wilk test, and homogeneity of variance across groups was determined using Levene’s test. Baseline characteristics, intubation characteristics, and outcome measures of the video laryngoscope group were compared with those of the direct laryngoscope group. Continuous data, which were all nonparametric, are presented as medians with first and third quartiles. Categorical variables are provided as numbers and percentages. Univariate analysis was done using a Chi-squared test or Fisher’s exact test (as applicable; categorical data) or Mann–Whitney *U* test (continuous data).

A binary logistic regression model was developed for the first attempt and overall successful endotracheal intubation. Direct laryngoscopy was used as reference group. The crude odds ratio (OR) is presented with 95% confidence interval.

## Results

**Fig. 1:**
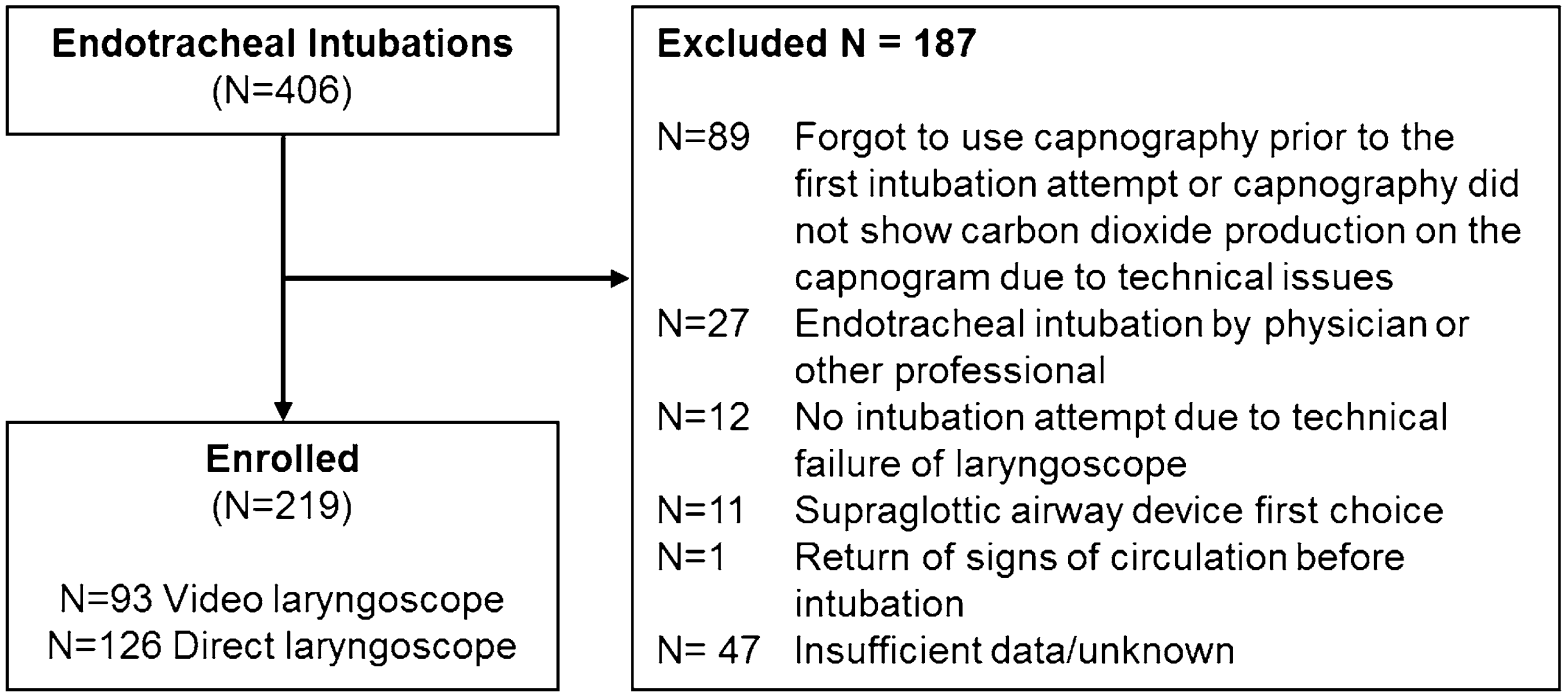
Flowchart of the study

During the study period, intubation was attempted in 406 patients. Of these, case report forms were completed for 219 patients (Fig. 1). Unexpected cancelation of the loan agreement by the manufacturer of the video laryngoscopes resulted in early termination of the study after these 219 patients were included. Ninety-three patients were intubated using the video laryngoscope and 126 using a direct laryngoscope. The patients had a median age of 70 (P_25_–P_75_ 50–78) years and the most common indication for intubation was a non-traumatic event (*n* = 197, 98.5%; Table 1). These characteristics did not differ between the two groups. In case the intubation was done by a nurse who had access to a video laryngoscope, this device was used.

**Table 1:** Patient and intubation characteristics and outcome of endotracheal intubation using a video or direct laryngoscope

Parameter	Overall (*N* = 219)		Video laryngoscopy (*N* = 93)		Direct laryngoscopy (*N* = 126)		*p* value
	*N*^a^		*N*^a^		*N*^a^		
Patient characteristics							
Age (years)	218	70 (59–78)	92	67 (59–75)	126	72 (58–79)	0.065
Event characteristics							
Nontraumatic	200	197 (98.5%)	84	84 (100%)	116	113 (97.4%)	0.266
Intubation and outcome details							
First attempt success	219	114 (52.1%)	93	53 (57%)	126	61 (48%)	0.221
Intubation time (seconds)	114	55 (38–67)	53	53 (36–64)	61	56 (38–69)	0.360
Overall success	219	157 (71.7%)	93	77 (83%)	126	80 (63%)	**0.002**
Intubation time	157	54 (38–71)	77	53 (37–70)	80	56 (39–72)	0.408
Backwards upwards rightwards pressure (BURP) used	217	63 (29.0%)	91	18 (20%)	126	45 (35.7%)	**0.015**
Stylet used	219	137 (62.6%)	93	53 (57%)	126	84 (66.7%)	0.159
Changes made between attempts	89		35		54		
Repositioned patient	89	60 (67%)	35	22 (63%)	54	38 (70%)	0.494
Stylet used	89	16 (18%)	35	11 (31%)	54	5 (9%)	**0.011**
BURP used	89	17 (19%)	35	5 (14%)	54	12 (22%)	0.418
Larger blade of tube	89	5 (6%)	35	0 (0%)	54	5 (9%)	0.152
Suction of mucous or debris from oral cavity	89	20 (22%)	35	10 (29%)	54	10 (19%)	0.305
Other	89	10 (11%)	35	4 (11%)	54	6 (11%)	1.000
Cause for failed intubation							
Anatomical issues	62	19 (31%)	16	5 (31%)	46	14 (30%)	1.000
Obese patient	62	12 (19%)	16	3 (19%)	46	9 (20%)	1.000
Presence of mucous or debris in oral cavity	62	32 (52%)	16	7 (44%)	46	25 (54%)	0.566
Corpus alienum	62	2 (3%)	16	1 (6%)	46	1 (2%)	0.453
Difficulty reaching patient	62	6 (10%)	16	1 (6%)	46	5 (11%)	1.000
Other	62	17 (27%)	16	6 (38%)	46	11 (24%)	0.338
Environmental issues	216	17 (7.9%)	91	6 (7%)	125	11 (9%)	0.617
Too much light	17	2 (12%)	6	2 (33%)	11	0 (0%)	0.110
Insufficient light	17	7 (41%)	6	2 (33%)	11	5 (45%)	1.000
Cold temperature	17	0 (0%)	6	0 (0%)	11	0 (0%)	N.A
Rainfall/precipitation	17	2 (12%)	6	1 (17%)	11	1 (9%)	1.000
Space limitations	17	4 (24%)	6	1 (17%)	11	3 (27%)	1.000
Other	17	3 (18%)	6	0 (0%)	11	3 (37%)	0.515
Alternative airway management	62		16		46		
Bag-valve-mask	62	15 (24%)	16	4 (25%)	46	11 (24%)	1.000
Supraglottic airway		47 (76%)		12 (75%)		35 (76%)	
HEMS assistance	214	12 (5.6%)	91	11 (12%)	123	1 (1%)	**< 0.001**
Return of spontaneous circulation (ROSC)	216	102 (47.2%)	93	40 (43%)	123	62 (50%)	0.336
Complications	212	4 (1.9%)	89	1 (1%)	123	3 (2%)	0.641
Technical problem	215	8 (3.7%)	92	4 (4%)	123	4 (3%)	0.727
Expected problems with intubation	214	89 (41.6%)	91	43 (47%)	123	46 (37%)	0.162
Expected positive effect of intubation device on success	184	121 (65.8%)	89	72 (81%)	95	49 (52%)	**< 0.001**

Categorical data are shown as *N* (%) and tested using Fisher’s exact test. Continuous data are shown as median (P_25_–P_75_) and tested using Mann–Whitney *U* test

*BURP* backwards upwards rightwards pressure, *HEMS* helicopter emergency services, *ROSC* return of spontaneous circulation

^a^These columns refer to the number of patients for whom data were available

Statistically significant *p*-values (*p* < 0.05) are indicated in boldface

### Success and time of endotracheal intubation

In 53 (57%) patients, the first video laryngoscopy-assisted intubation attempt was successful. This was similar to the first attempt success in the direct laryngoscopy group (*n* = 61; 48%; *p* = 0.221; Table 1). Overall success, on the other hand, was higher in the video laryngoscopy group (*n* = 77, 83%) than in the direct laryngoscopy group [(*n* = 80, 63%); *p* = 0.002). The binary logistic regression analysis showed a crude OR for overall success of 2.767 [95% confidence interval (CI) 1.446–5.297; *p* = 0.002] in favor of video laryngoscopy.

The median time needed to achieve first attempt and overall success was 53 s for the video laryngoscopy group. This was not statistically different from the 56 s needed when using a direct laryngoscope (Table 1).

### Other intubation characteristics

Backwards upwards rightwards pressure (BURP) was used in fewer patients in the video laryngoscopy group (*n* = 18, 20%) than in the control group [(*n* = 45, 36%); *p* = 0.015). Stylet use in both groups did not differ.

For 89 patients requiring a second intubation attempt, the four most common changes in between the two attempts were repositioning of the patient (*n* = 60), suction of mucous or debris from the oral cavity (*n* = 20), use of BURP (*n* = 17), and use of stylet (*n* = 16). Of these changes, only stylet use was reported more often for the video laryngoscopy group (*n* = 11, 31%) than for the control group (*n* = 5, 9%; *p* = 0.011).

For the 89 patients requiring a second intubation attempt, the ambulance nurse reported patient-related or environmental reasons for the initial failure in 62 and 17 cases, respectively. The most commonly reported patient-related reasons were the presence of mucous or debris in the oral cavity (*n* = 32), anatomical issues (*n* = 19), and patient obesity (*n* = 12). The most commonly reported environmental reasons were insufficient light and space. None of the patient-related or environmental reasons for intubation failure differed statistically significantly between the two groups.

In the 62 patients in whom intubation was not successful after two attempts, alternative strategies were used; in 47 (76%), a supraglottic airway was chosen. This was similar in both groups.

### (Device) complications

In the entire group, four complications were encountered. In one patient in the direct laryngoscopy, group traces of blood were found on the tip of the tube and the supraglottic airway device. In a second patient in this group, an abscess ruptured during intubation. The tube of one patient filled with blood or sputum after placement using a direct laryngoscope. The only complication in the video laryngoscopy group was the finding of blood at the vallecula, probably due to maneuvering of the laryngoscope.

The ambulance nurses also reported four technical problems in each group. Three direct laryngoscopes had light issues and one showed a defect of the laryngoscope. In two occasions, the video laryngoscope showed condensation of the camera lens, blurring the view on the screen. In another case, the ambulance nurse was unable to connect the blade to the laryngoscope. Insufficient light was reported in a fourth video laryngoscopy failure.

### Opinion of the ambulance nurses

In 89 intubations, the ambulance nurses judged the intubation as potentially difficult; no association with the intubation device was noted. In the majority of intubations, they reported that the device used had a positive effect on the intubation success. This was reported more often in the video laryngoscopy group (*n* = 72, 81%) than in the direct laryngoscopy group (*n* = 49, 52%; *p* < 0.001).

## Discussion

In this prospective observational study of patients undergoing prehospital endotracheal intubation by ambulance nurses in the Netherlands comparing the use of video laryngoscopy with direct laryngoscopy, no statistically significant difference in first attempt success rate for endotracheal intubation was found between video and direct laryngoscopy. The overall success rate for endotracheal intubation, however, was positively impacted by the use of video laryngoscopy.

Video laryngoscopy has gained much attention over recent years, as it is hypothesized to allow for easier visualization of the vocal cords and subsequent tube placement. Indeed, a 2017 Cochrane review showed that video laryngoscopy increased easier laryngeal views (OR 6.77, 95% CI 4.17–10.98) and decreased the number of failed intubations (OR 0.32, 95% CI 0.13–0.75) [5]. However, many included studies were performed under elective circumstances and the decreased chance of failed intubation was only apparent in experienced users. Another systematic review and meta-analysis by Savino et al*.* specifically looked at prehospital intubations by both physicians and non-physicians [6]. Meta-analysis of data about non-physician intubation was performed on data derived from four retrospective, observational studies [7–10]. While two of these studies did not observe a statistically significant difference between video laryngoscopy and direct laryngoscopy with regard to overall intubation success, meta-analysis of these data marginally favored video laryngoscopy [relative risk (RR) 2.2; 95% CI 1.00–5.02]. Interestingly, this meta-analysis found that video laryngoscopy did positively contribute to the first-pass success rate in non-physicians (RR 1.83, 95% CI 1.18–2.84). Our study adds to the current data by showing that video laryngoscopy positively impacts on the overall successful intubation rate by non-physicians in the prehospital environment.

In the current study, both the first-pass success rate (52%) and the overall success rate (72%) for intubation were quite low. These numbers are similar to those found in an earlier study from the Netherlands, describing a first attempt success rate of only 48% when direct intubation was performed by Dutch ambulance nurses [11]. When comparing these first attempt and overall success rates for intubation to other available literature, the numbers for the Netherlands are somewhat disappointing. In a systematic review published in 2017, which included 38 studies on prehospital intubation, the pooled crude intubation success rates for non-physicians were 92% (range 62–100%) and the reported first attempt success rate was 70% (range 63–97%) for non-physicians [2]. Caution is warranted when comparing different studies on this subject, however, as many factors may have contributed to a high rate or low rate of successful intubations. First, the level of training and experience of providers and the number of cases requiring advanced airway management which they are exposed to annually may significantly differ between prehospital emergency medical services. Indeed, Dutch ambulance nurses are estimated to perform somewhere between five and ten intubations annually, a much lower number than needed to keep intubation skills alive; A review article by Buis et al. concluded that a minimum of 50 intubations per year would be required to keep skills up to date [1]. Second, as Dutch ambulance nurses are only allowed to intubate patients with a GCS-score of three (excluding patients with a neurological origin of their loss of consciousness) without the use of paralytic drugs, while in other countries, services allow for drug-assisted intubation by non-physicians. Moreover, it is likely that most intubations in this study were performed on patients in cardiac arrest receiving chest compressions at the moment of laryngoscopy, which is known to add more complexity to the procedure.

Given these low success rates, there is ongoing debate about whether or not endotracheal intubation should be the method of first choice in prehospital airway management by non-physicians, since a poorly executed endotracheal intubation attempt may impact quality of resuscitation and even survival [12]. It has, therefore, been suggested that supraglottic airway devices (which are much easier to insert) should be the device of first choice in prehospital airway management by non-physicians [4]. Unfortunately, two large systematic reviews and meta-analyses did not find better survival or neurologic outcome in patients with out-of-hospital cardiac arrest whose airway was managed by a supraglottic airway when compared with endotracheal intubation [13, 14]. In addition, a well-executed RCT by Benger et al. randomizing 9296 patients with out-of-hospital cardiac arrest between endotracheal intubation or a supraglottic airway device did not show any difference in 30 day outcome [15]. A major disadvantage of a supraglottic airway first approach would be a further loss of exposure to endotracheal intubation for ambulance nurses and paramedics resulting in even lower success rates for patients in whom a supraglottic airway device fails to facilitate adequate ventilation.

This study has several limitations. First, the study had to be terminated early because of unexpected cancelation of the loan agreement by the new manufacturer and distributor of the video laryngoscopes. The study did, therefore, not meet the calculated power requirements potentially increasing the risk of a type-2 error with regard to the difference in first-pass effect. Second, results may have been confounded by the use (or lack thereof) of stylets in both the direct laryngoscopy group as well as the video laryngoscopy group. The overall use of stylets was low, which may have contributed to low first-pass percentages. Moreover, stylets were used more often in the video laryngoscopy after a failed first attempt, possibly skewing results.

## Conclusions

Data of the current study show that the use of video laryngoscopy resulted in a higher overall success percentage, but not in a higher first attempt success percentage in prehospital intubation by Dutch emergency medicine services. Video laryngoscopy should, therefore, be the method of first choice for non-physician prehospital intubation in comparable settings.
